# COVID-19: A Review of Emerging Preventative Vaccines and Treatment Strategies

**DOI:** 10.7759/cureus.8206

**Published:** 2020-05-20

**Authors:** Kashmala Khan, Francis Dimtri, Carlos Vargas, Salim Surani

**Affiliations:** 1 Internal Medicine, Corpus Christi Medical Center, Corpus Christi, USA; 2 Cardiology, Corpus Christi Medical Center, Corpus Christi, USA; 3 Internal Medicine, University of North Texas, Dallas, USA

**Keywords:** sars-cov-2, covid-19, coronovirus, mrna-based vaccine, vaccine development, clinical trials

## Abstract

COVID-19, which was first detected in the Hubei province of China, has become a global phenomenon. The effects and devastation on both health and economy have been global. At present, there is a substantial amount of research being done to discover suitable treatment modalities. Efforts have been made on the development of potential efficacious vaccines. The development of a vaccine can be complex, expensive as well as time-consuming. Currently, various ongoing clinical trials are in progress that are investigating either pharmacologic therapies or vaccines against this virus. We, in this brief review have tried to address the process and current development efforts of vaccine in progress.

## Introduction and background

Infecting millions and causing tens of thousands of deaths worldwide, the corona virus disease 2019 (SARS-CoV-2) or COVID-19 pandemic has become one of the most devastating outbreaks. It has been declared a public emergency by the WHO (World Health Organization) [1]. As the number of infected cases and mortality rises, fear and unrest have grown during these unprecedented times. There is no definitive curative, preventative therapy or any set guidelines for physicians to help tackle this virus, while they sail in this unchartered territory. Remdesivir has shown some initial promises, whereas caution has been advised against the use of chloroquine and related drug hydoxychloroquine due to possible lack of efficacy and safety concerns [2]. The labile nature of events that have ensued over the past few months has created a disconcerting panic and fear. One core aspect of dealing with the resilient pathogens in the past has been the coveted vaccine. Immunization has helped save millions of lives from preventable and fatal diseases. In this review, we will briefly discuss the highly sought vaccine for COVID-19, the dynamics, and the setbacks that can entail in developing such a vaccine.

## Review

The coronaviruses (CoVs) are enveloped positive sense, single-stranded RNA viruses. They belong to the family of viruses known as Coronaviridae, subfamily Coronavirinae, and the order Nidovirales [3]. This order further consists of four genera: alphacorona, betacorona, gammacorona, and deltacorona. Bats and rodents are found to be sources of the alphacorona and betacorona, whereas deltacorona and gammacorona have frequently found to have avian species as the source [4]. Of the above mentioned, alphacorona and betacorona have been known to infect mammals. The new COVID-19 is part of the betacoronaviruses [4]. Well-recognized CoVs that cause infections in humans are the SARS (severe acute respiratory syndrome) CoV, and MERS (Middle Eastern respiratory syndrome) CoV. That is why the CoV causing COVID-19 is also called SARS-CoV-2 and is believed to have evolved from the virus that was found in bats. The CoV that has caused COVID-19 has been shown to have 89% similarity in its genome with the bat SARS-like-CoVZXC21, and 82% similarity with the human SARS-CoV [1].

The virus enters its host membrane via the S protein through the angiotensin converting enzyme 2 receptor on the host membrane [5]. It then releases its genome into the host cytoplasm, the replicase gene is translated from the viral genomic RNA. This is followed by viral RNA synthesis which codes for both genomic and sub-genomic RNA. The sub-genomic RNA in turn acts as an mRNA for accessory and structural genes. That along with the structural proteins are translated and enter the endoplasmic reticulum to be assembled and released [5]. Multiple ongoing clinical trials are currently focusing on pharmacologic therapy, including repurposed and investigational drugs to treat active COVID-19 in adults. Therapies that have been studied so far target specific viral components or a specific phase in the infective process. Chloroquine, hydroxychloroquine, camostat mesylate, and arbidol interfere with viral receptor attachment [6]. Chloroquine and hydroxychloroquine also affect the endocytic pathway of the virus. Drug treatments that target nonstructural proteins (e.g. RNA-dependent RNA polymerase) include ribavirin, remdesivir, and favipiravir. The viral replication phase is also disrupted by lopinavir and darunavir that inhibit proteolysis of viral polypeptides to nonstructural proteins [6]. Lastly there are drugs that reduce the immune response to the virus and inhibit the inflammatory pathway which prevents further organ damage. Tocilizumab and sarilumabantiI act as IL-6 receptor antagonists, prevent further propagation of the IL-6 signaling, and help neutralize the host immune response [3, 6-8].

About 2% of the population are carriers and roughly 5%-10% of viral respiratory infections are caused by CoVs. The goal of a vaccine against a viral pathogen is to prevent the spread, frequency, and severity of the disease. There are several mechanisms by which this is achieved. The first is inducing antibodies to the surface glycoproteins or capsid proteins. The antibodies detect the virus and attack it and cause neutralization before the infective process propagates. The second and less common mechanism is T-cell mediated destruction of virus-infected cells. This helps control rather than prevent the disease [7].

Vaccines against viruses can be live attenuated, inactivated, or recombinant and peptide-based [8]. A live attenuated vaccine introduces the virus into the body, but it has decreased virulence and so causes a much milder infection. They elicit a response from the immune system by producing memory cells that are activated later on and protect against future infections. Some viruses are rendered “inactive” by chemical or physical means because they have an increased risk of virulence and cannot be attenuated. They do not stimulate the immune system as well as a live attenuated vaccine and are, therefore, considered weaker and less effective. Recombinant vaccines use a specific part or subunit of a virus or the whole virus without the key virulent segments. These too are not as efficacious [8].

Viruses mutate and evolve spontaneously to increase their chance of survival. Genetic mutation corresponds to greater genomic variations. Various viruses have different mutation rates. DNA viruses mutate slower than RNA viruses and negative sense viruses have slower mutation rates when compared to positive sense viruses [9]. This is one of the reasons why it is challenging to target a vaccine to an ever-changing and constantly evolving genome. Two RNA-based vaccines have been studied. The first is a conventional mRNA-based vaccine which encodes only the antigen that is to be presented to the immune system. The second is a self-amplifying mRNA-based vaccine which is based on the genome of positive strand RNA viruses. This self-amplifying mRNA vaccine encodes for the antigen and also the genomic material required for the replication of the virus, which does not include genes for the structural proteins (Table 1). All of this can “amplify” and increase protein expression to the immune system [10].

**Table 1:** Summary of different types of mRNA-based vaccines [10].

Type of mRNA vaccine	Mechanism of action	Comments
Conventional	Genes encode only for the target antigen	The antigen expression is only transient, so the immune response generated is weaker
Amplifying	Genes encoding the RNA are intact but instead if structural proteins, the genes encode for the target antigen	Higher antigen expression, although delayed effect. The immune response elicited lasts longer

 For a vaccine to be accepted and widely used, it needs to be evaluated for both safety and efficacy. In addition, there is also an extensive development process. Guidelines are provided by the World Health Organization (WHO), European Medicine Agency (EMA), and the United States Food and Drug Administration (FDA) [11]. The whole process takes approximately 10-15 years on average and costs roughly one billion US dollars. There are many trials that may fail in the developmental phase. Development starts with nonclinical animal trials. Once valuable data is collected, manufacturers move on to clinical trials in humans. Phase I trials which are randomized, double-blind, placebo-controlled, single-center studies consist of testing the vaccine in healthy, immunocompetent adults. In phase II the vaccine is used in the target population, and developers look into dosage, scheduling, immune response, and multiple variables. One of the most important features of a phase II trial is evaluating the safety profile of the vaccine. Phase III is similar to phase II but the former uses a much larger target population which is essential for marketing approval (Table 2) [12].

**Table 2:** Summary of different phases of clinical trials [12].

Phase	Goal	Study population	Study design
I	To evaluate the safety, adverse effects and immune response to the vaccine	Healthy immunocompetent adults	Open-labeled nonrandomized
II	To evaluate dosing, scheduling of the vaccine	Subjects from target population	Randomized controlled
III	For registration and approval of the vaccine	Larger scale, thousands of subjects from target population	Randomized controlled

Since the outbreak of SARS-CoV-2, researchers have been working tirelessly to develop an effective vaccine in real-time that would help reduce the current spread of this disease as well as prevent future infections. In the United States, several trials are currently taking place for this exact purpose. The National Institute of Allergy and Infectious Disease (NIAID) has started a nonrandomized open- labeled dose ranging clinical trial in healthy adult males and nonpregnant females. This phase I trial is using a mRNA-based vaccine that encodes for a stabilized spike (S) protein of SARS-CoV-2. Another phase I trial is by Inovio Pharmaceuticals, which is an open-labeled, nonrandomized trial that is studying the immunologic profile and tolerability of a preventative vaccine against SARS-CoV-2 [13]. The Shenzhen Geno-Immune Medical Institute is currently recruiting subjects for two clinical trials. The first is an open-labeled trial with an artificial genetically modified antigen presenting cell vaccine against SARS-CoV-2. The second is a phase I/II, multi-center open-labeled trial that is evaluating the safety of injecting an antigen-specific cytotoxic T-cell vaccines among healthy subjects. There are two trials that are looking into the use of the BCG vaccine for the prevention of SARS-CoV-2. The previous studies have suggested the vaccine to have some unexplained benefits in various respiratory infections. The first trial is a placebo-controlled adaptive multi-center randomized controlled trial UMC (University Medical Center) Utrecht in which participants are given either the BCG vaccine or placebo in a 1:1 ratio. The second trial by Murdoch Children’s Research Institute is a phase III, two group, multi-center, open-labeled, randomized controlled trial. Other trials include the phase II, randomized, double-blinded, and placebo-controlled clinical trial by the Institute of Biotechnology, Academy of Military Medical Sciences, PLA of China which is using an adenovirus vector which encodes for the S (spike) protein of the SARS-CoV-2. The phase I, randomized, observer-blind, placebo-controlled trial by Symvivo Corporation is using plasmids with genetic material that code for the S protein, but this vaccine is delivered orally. Oxford University is conducting a phase I/II single-blinded, randomized, placebo-controlled, multi-center study to test the safety of an intramuscularly administered against SARS-CoV-2. CanSino Biologics Inc. is conducting a single-center, open-labeled, phase I trial that is using an adenovirus vector for their vaccine (Table 3) [13].

**Table 3:** List of current clinical trials investigating vaccines against COVID-19 [13].

Company	Phase	Country	Design	Mechanism	Targeted completion date
Shenzhen Geno-Immune Medical Institute	I	China	Open label, nonrandomized	aAPC vaccine	31-Dec-24
Shenzhen Geno-Immune Medical Institute	II/III	China	Open label, nonrandomized multicenter	antigen-specific cytotoxic T-cell vaccine	31-Dec-24
UMC Utrecht	III	Netherlands	Randomized controlled trial	BCG vaccine	25-Dec-20
CanSino Biologics Inc.	I	China	Single-center, open label, nonrandomized	Adenovirus type 5 vector	20-Dec-22
Murdoch Childrens Research Institute	III	Australia	Open label randomized controlled trial	BCG vaccine	30-Mar-22
Symvivo Corporation	I	Canada	Randomized, observer-blind, placebo-controlled trial	bacTRL-Spike vaccine	31-Dec-21
University of Oxford	I/II	United Kingdom	Single-blinded, randomized, placebo-controlled, multi-center study	Intramuscular biologic vaccine	May 2010-Now being revised to 9/2020
Institute of Biotechnology, Academy of Military Medical Sciences, PLA of China	II	China	Randomized, double-blind, placebo-controlled	Adenovirus type 5 vector	31-Jan-21
National Institute of Health, Emory University, Kaiser Permanente	I	United States	Open label, dose-ranging study	mRNA-1273	1-Jun-21
Inovio Pharmaceuticals	I	United States	Open label, nonrandomized trial	intradermal (ID) injection followed by electroporation	Nov-20
Medical University of Vienna		Austria	Observational	Assess occult immunization. Measure IGM, IGG, IGA agansr Sar-	44297
Cairo University	III	Egypt	Randomized	MMR vaccine	Nov-20
Faculty of Medicine Ain Shams University Research Institute	III	Egypt	Randomized	BCG vaccine	Dec-20
Harvard/Texas A&M Univ/Baylor & three more	IV	USA	Randomized	BCG vaccine	Nov-21

## Conclusions

Although containment and mitigation efforts are being undertaken globally, despite that morbidity, mortality, and economic toll of SARS-CoV-2 remain high. This has led physicians, researchers, and pharmaceutical companies to work tirelessly towards an effective preventative vaccine and treatment strategies. Viral illnesses have historically been difficult to treat due to the rapid, dynamic mutation the viruses undergo. This evolutionary response helps in genetic variation and increases survival, while making it challenging to produce a vaccine tailored for any specific virus. There have been great strides in recent months with many upcoming and promising clinical trials worldwide. Lessons from SARS and Ebola have prepared laboratories globally to attempt to develop an efficacious and safe vaccine in a short period. While most trials are actively recruiting healthy volunteers, it is still unclear to what extent the vaccine will benefit those most at risk currently. Also, not having any effective proven therapy makes the situation complicated as healthcare providers continue their journey of saving the population infected with SARS-CoV-2 worldwide proving once again patients come first.
